# Exploring the association between BMI and dental caries in 6–9-year-old children in Damascus, Syria: a cross-sectional study

**DOI:** 10.1038/s41405-025-00383-z

**Published:** 2025-12-01

**Authors:** Alaa Ashour, Lana Alshayeb, Mayssoon Dashash

**Affiliations:** Pediatric Dentistry Department, Faculty of Dentistry, Damascus University, Damascus, Syria

**Keywords:** Dental public health, Paediatric dentistry

## Abstract

**Aim:**

To investigate the prevalence of dental caries and its association with Body Mass Index (BMI) among Syrian schoolchildren aged 6–9 years in Damascus.

**Materials and methods:**

A cross-sectional study was conducted among 462 schoolchildren selected through multi-stage cluster sampling. Data were obtained using a structured questionnaire and clinical examinations based on WHO criteria. BMI was assessed according to standard protocols. Associations between dental caries and sociodemographic, dietary, oral hygiene factors, and BMI were analyzed using SPSS v25, with statistical significance set at *P* < 0.05.

**Results:**

The prevalence of dental caries was 85.5%, with no significant differences by gender or age. Caries was significantly more frequent among children in public schools (87.7%; *P* = 0.042) and those from low socioeconomic backgrounds (95.7%; *P* < 0.001). Irregular fruit and vegetable intake and higher sugar consumption were associated with increased caries risk. BMI also showed a significant association with caries (*P* < 0.001), with overweight children exhibiting the highest prevalence (97.1%), although multivariable logistic regression confirmed BMI as an independent predictor with no difference between obese children and other classifications.

**Conclusion:**

Dental caries is highly prevalent among Syrian children aged 6–9 years and shows significant associations with socioeconomic disadvantage, unhealthy dietary habits, poor oral hygiene, and increased BMI. Nevertheless, due to the cross-sectional design of the study, these associations do not establish a causal relationship between BMI and dental caries. Despite this limitation, the findings underscore the urgent need for integrated public health strategies to address both oral health and childhood obesity.

## Introduction

Dental caries remains a significant public health concern, particularly in low- and middle-income countries, despite various preventive measures [1–3]. Globally, over 600 million children are affected by untreated dental caries [4]. The development of this oral health condition is influenced by multiple factors, including biofilm accumulation, dietary habits, saliva composition, individual behaviors, and socioeconomic status [5], and it can lead to pain, abscesses, learning difficulties, behavioral issues, and limitations in parental activities [6].

In Syria, studies have reported high dental caries prevalence among children, ranging from 61% (Dashash and Blenkhorn) to 79.1% (Ballouk and Dashash) and up to 91% (Alshayeb and Dashash) across different age groups [7–9]. However, these studies did not specifically examine children aged 6–9 years, highlighting a gap that the present study aims to address.

Obesity is a major public health concern affecting children and adolescents worldwide [10, 11], and its prevalence has risen substantially over recent decades, prompting the World Health Organization to declare it a global epidemic [12]. In Syria, despite eight years of civil war, a study by Zamlout et al. reported increasing obesity rates among children aged 2–20 years—4.5% in males and 3.66% in females—while overweight prevalence reached 20.1% in males and 19.54% in females, figures higher than those observed in neighboring countries [13]. Obesity in children is associated with numerous physical health consequences, including diabetes and hypertension, as well as behavioral and mental health challenges [14, 15].

Body Mass Index (BMI), although not a direct measure of body fat, is widely used for classifying individuals as underweight, normal weight, overweight, obese, or severely obese due to its simplicity and ease of calculation [16].

Overweight and dental caries are prevalent chronic health issues that share multiple risk factors, including dietary habits, food availability, sugar consumption, genetic predispositions, cultural influences, and socioeconomic conditions [17, 18]. Growing attention has focused on the potential link between these conditions [17]; however, this association does not imply a direct causal relationship, but rather reflects the clustering of shared risk factors within affected populations [19]. The relationship between obesity and dental caries remains controversial, with inconsistent findings reported across studies [15].

For example, Mohajeri et al. found no significant association between BMI and dental caries [20], a conclusion similarly reported by Macek et al. in a study of American children [21]. In contrast, a study of 27,333 New Zealand children revealed that there was a significant correlation between BMI and dental caries, particularly among children of European ethnicity [22]. Such variations in findings may be attributed to differences in obesity definitions, age groups studied, and ethnic background [23].

Although Jaghasi et al. assessed the dietary habits of Syrian children and reported high sugar consumption and poor oral hygiene, their study did not examine BMI as a risk factor for dental caries [24]. Moreover, many epidemiological studies on dental caries have overlooked the potential role of BMI in caries prevalence across different age groups [8, 9, 25, 26].

Therefore, this study specifically addresses a critical gap in the existing literature by investigating the prevalence of dental caries and its association with Body Mass Index (BMI) among Syrian children aged 6–9 years in Damascus. By focusing on this narrow age group, the study provides novel insights into the interplay between nutritional status, dietary habits, oral hygiene practices, and caries risk.

## Materials and methods

This descriptive, cross-sectional study examined the prevalence of dental caries and its association with Body Mass Index (BMI) among Syrian schoolchildren aged 6–9 years in Damascus during the 2023–2024.

All study procedures complied with current regulations and ethical standards, including the Declaration of Helsinki [27]. Ethical approval was obtained from the Scientific Research Committee at the University of Damascus, Syria (reference number 2952), alongside authorization from the Minister of Education. Permission was also obtained from school principals, and informed consent was collected from parents or caregivers. This study was reported in accordance with the Strengthening the Reporting of Observational Studies in Epidemiology (STROBE) guidelines for observational studies.

To ensure representativeness and minimize selection bias, a multi-stage cluster sampling method was employed. In the first stage, Damascus was divided into nine administrative areas based on the Ministry of Health classification. In the second stage, a list of all elementary schools within each area was compiled, and three schools were randomly selected using a simple lottery method, in which each school was assigned a number and numbers were drawn to determine the schools included. In the third stage, within each selected school, classes were randomly chosen, and all students aged 6–9 years in those classes were invited to participate. However, the number of participants was not equal across sectors or between public and private schools.

The sample size was primarily estimated based on the expected prevalence of dental caries, which was considered the main outcome of interest. Moreover, the sample size was calculated using Cochran’s formula (1963) [28], with a 95% confidence level. The calculation was based on (Z = 1.96), a 5% margin of error (e = 0.05), and an estimated population proportion of 50%, resulting in a minimum of 384 participants to ensure 90% study power. To improve reliability, Caregivers of 502 children were invited to provide consent for participation, of whom 462 agreed, yielding a participation rate of 92% and a refusal rate of ~8%. The primary hypothesis of the study was that dental caries is highly prevalent among children aged 6–9 years in Damascus, while the secondary hypothesis was that there is a significant association between body mass index (BMI) and dental caries prevalence.

The purpose and procedures of the study were explained to children and their parents, ensuring confidentiality and voluntary participation.

The inclusion criteria encompassed Syrian children aged 6–9 years who were lifelong residents of Damascus, had no relevant medical history, and provided signed consent.

Exclusion criteria included children wearing orthodontic appliances, developmental dental defects, smoking habits, diagnosed mental or physical disabilities, or uncooperative behavior.

The study was implemented in two distinct phases. The first phase comprised the administration of a structured and pretested questionnaire, which was completed by parents. This instrument was designed to capture information across three domains: sociodemographic characteristics (including the child’s age, gender, school type, parental education, and socioeconomic status), oral hygiene practices (such as frequency of tooth brushing, flossing, and the use of mouth rinses), and dietary habits (including daily sugar intake, fruit consumption, and vegetable consumption).

Prior to its administration, the questionnaire was critically reviewed by a panel of experts in pediatric dentistry and public health to ensure face and content validity, thereby confirming the clarity, relevance, and comprehensiveness of the items.

To further establish reliability, a pilot study was conducted with 50 children, and internal consistency testing was performed on the dietary and oral hygiene constructs. The analysis demonstrated a Cronbach’s α coefficient of 0.88, reflecting high internal consistency and supporting the robustness of these measures for subsequent analyses.

The second phase was clinical. The clinical examination consisted of a dental exam and anthropometric measurements.

Dental caries were assessed using World Health Organization (WHO) diagnostic criteria and the DMFT/dmft indices [29]. It should be noted that this approach may underestimate the presence of early enamel lesions, as only cavitated lesions reaching the dentin are recorded.

A single examiner (A.A.) performed examinations in a quiet classroom using standardized instruments and infection control measures. A headlamp provided illumination, and the child was seated on a standard chair. The examiner used sterile mouth mirrors, WHO dental probes, disposable gloves, and a surgical face mask. Additional sterile materials, such as cotton rolls and gauze, were also used during the examination.

Lesions were recorded only when enamel breaks or underlying caries shadows were visible, with uncertain cases classified as sound [30].

Height was measured using a stadiometer with children standing upright, head in the Frankfurt plane, arms relaxed, feet apart, and heels touching the vertical rod, recorded to the nearest millimeter. Weight was measured using portable electronic scales accurate to 100 g, following protocols from Zamlout et al. [13].

Body Mass Index (BMI) was calculated as weight [31] divided by height squared (m²) [32]. Overweight and obesity cut-offs were based on the growth percentiles for children and adolescents defined by Zamlout et al. [13].

classifying the Body Mass Index as thinness (thinness <9%, normal (9–75%), overweight 75–97.7%, and obesity >97.7%).

Statistical analyses were conducted using SPSS version 25. Descriptive statistics summarized frequencies and percentages. Chi-square *tests* examined the relationship between dental caries and study variables. Variables showing significance in preliminary tests were subsequently entered into *multivariate logistic regression models* (Forward: WALD method). A *P-value* of less than 0.05 was considered statistically significant.

### Ethics approval and consent to participate

Ethical approval for this study was obtained from the Scientific Research Committee at Damascus University, Syria (IRB No. UDDS-1593-07052023/SRC-2952). In addition, official authorization was granted by the Ministry of Education. Permission was secured from the principals of all participating schools, and written informed consent was obtained from the parents or legal guardians of all participating children.

## Results

Among the 462 children included in the study (46% males, 54% females), dental caries was highly prevalent, affecting 85.5% of participants with a mean Decayed, Missing, and Filled permanent Teeth (DMFT) + decayed, missing, and filled primary Teeth (dmft) score of 4.53 ± 2.79. The prevalence of dental caries did not differ significantly between sexes or across age groups (Table 1).

**Table 1:** Descriptive distribution and association of demographic, socioeconomic, dietary, and oral hygiene factors with dental caries prevalence among children.

		Total		Dental caries				*P*-value
				Yes		No		
		*N*	%	*N*	%	*N*	%	
Gender	Male	214	~46	183	85.5	31	14.5	0.993
	Female	248	~54	212	85.5	36	14.5	
Age	6–<7	160	34.6	137	85.6	23	14.4	0.988
	7–<8	148	32.0	126	85.1	22	14.9	
	8–9	154	33.3	132	85.7	22	14.3	
School type	Public	326	70.6	286	87.7	40	12.3	0.042^*^
	Private	136	29.4	109	80.1	27	19.9	
Family socio-economic status	Low	235	50.9	225	95.7	10	4.3	0.001^*^
	Moderate	128	27.7	104	81.3	24	18.8	
	High	99	21.4	66	66.7	33	33.3	
Father’s education level	Low	97	21.0	82	85.5	15	15.5	0.651
	Moderate	197	42.6	166	84.3	31	15.7	
	High	168	36.4	147	87.5	21	12.5	
Mother’s education level	Low	71	15.4	57	80.3	14	19.7	0.178
	Moderate	211	45.7	178	84.4	33	15.6	
	High	180	39.0	160	88.9	20	11.1	
Mother’s work	Yes	190	41.1	167	87.9	23	12.1	0.231
	No	272	58.9	228	83.8	44	16.2	
Frequency of daily fruit consumption	Never	260	56.3	236	90.8	24	9.2	0.001^*^
	Once a day	173	37.4	148	85.5	25	14.5	
	Twice or more a day	29	6.3	11	37.9	18	62.1	
Frequency of daily vegetable consumption	Never	244	52.8	219	89.8	25	10.2	0.020^*^
	Once a day	210	45.5	169	80.5	41	19.5	
	Twice or more a day	8	1.7	7	87.5	1	12.5	
Frequency of daily sugar consumption	Never	139	30.1	89	64.0	50	36.0	0.001^*^
	Once a day	135	29.2	126	93.3	9	6.7	
	Twice or more a day	188	40.7	180	95.7	8	4.3	
Daily toothbrushing	Never	193	41.8	176	91.2	17	8.8	0.013^*^
	Once a day	134	29.0	109	81.3	25	18.7	
	Twice	135	29.2	110	85.5	25	18.5	
Using dental floss or an oral rinse	Yes	22	4.8	18	81.8	4	18.2	0.543
	No	440	95.2	377	85.7	63	14.3	
Body Mass Index	Under-weight	24	5.2	15	62.5	9	37.5	0.001^*^
	Normal	294	63.6	244	83.0	50	17.0	
	Over-weight	104	22.5	101	97.1	3	2.9	
	Obesity	40	8.7	35	87.5	5	12.5	

Chi-square test.

*N* Number of cases, *%* Percentage.

^*^a significant difference.

The prevalence of caries was notably higher among children attending public schools compared to those in private schools. Socioeconomic status emerged as a strong determinant, with children from low-income families exhibiting the highest prevalence, whereas those from high-income families had the lowest (*P-value* = 0.001) (Table 1).

Dietary habits were closely associated with caries experience. Lower consumption of fruits and vegetables was linked to an increased risk, while higher intake of these foods corresponded with reduced caries prevalence. In contrast, frequent sugar consumption substantially elevated the likelihood of dental caries (*P-value* = 0.001) (Table 1).

Oral hygiene practices further influenced caries prevalence. Children who did not brush their teeth daily exhibited higher rates of caries, whereas the use of dental floss or mouth rinses was not significantly associated with caries (Table 1).

**Fig. 1:**
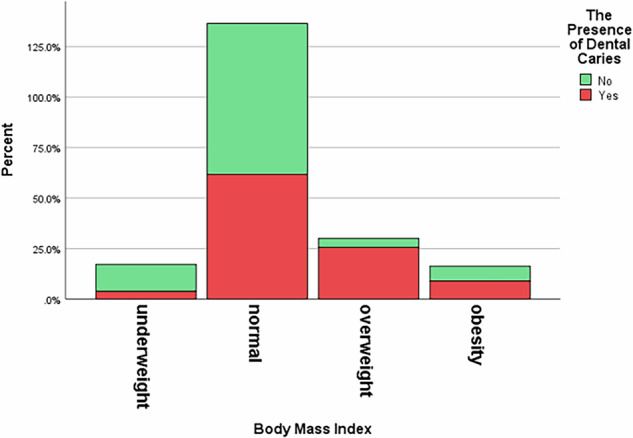
Bar chart for body mass index vs dental caries.

Body mass index was also related to dental health, with overweight children showing a higher prevalence of caries compared to their peers (*P-value* = 0.001) (Table 1) (Fig. 1).

Multivariable logistic regression confirmed that several factors were significant predictors of dental caries. Children from low socioeconomic backgrounds had 19 times higher odds of experiencing caries compared to those from high-income families (OR = 19.096; 95% CI: 7.076–51.533; *P-value* = 0.001) (Table 2).

**Table 2:** The fifth model was developed using multivariable logistic regression to analyze the explanatory variables associated with dental caries.

		OR^a^	*P-value*	Reference group
Family socio-economic status			0.001^b^	High
	Low	19.096 (7.076–51.533)	0.001^b^	
	Moderate	1.991 (0.896–4.424)	0.091	
	High	1^c^		
Frequency of daily fruit consumption			0.001^b^	Twice or more a day
	Never	20.282 (5.885–69.897)	0.001^b^	
	Once a day	12.570 (3.537–44.674)	0.001^b^	
	Twice or more a day	1^c^		
Frequency of daily vegetable consumption			0.012^b^	Twice or more a day
	Never	0.452 (0.022–9.358)	0.608	
	Once a day	0.154 (0.007–3.192)	0.227	
	Twice or more a day	1^c^		
Frequency of daily sugar consumption			0.001^b^	Twice or more a day
	Never	0.117 (0.046–0.297)	0.001^b^	
	Once a day	0.837 (0.274–2.559)	0.755	
	Twice or more a day	1^c^		
Body Mass Index			0.021^b^	Obesity
	Under-weight	0.259 (0.041–1.638)	0.151	
	Normal	1.344 (0.300–6.021)	0.699	
	Over-weight	3.452 (0.546–21.831)	0.188	
	Obesity	1^c^		

Values are presented as numbers with 95% confidence intervals (CI).

^a^*OR* Odds Ratio. OR > 1 indicates higher odds of outcome compared to reference; OR < 1 indicates lower odds.

^b^A significant difference.

^c^Indicates the reference.

Similarly, children who never consumed fruits had 20 times higher odds of caries than those consuming fruits twice or more daily (OR = 20.282; 95% CI: 5.885–69.897; *P-value* < 0.001). High sugar intake was also a strong predictor: children who never consumed sugar had lower odds of caries (OR = 0.117; 95% CI: 0.046–0.297; *P-value* = 0.001) compared to those consuming sugar twice or more daily (Table 2).

Obese children were chosen as the reference group to allow comparison of caries risk across BMI categories relative to the group typically considered at highest health risk. Overweight children showed higher odds of dental caries compared to obese children (OR = 3.452; 95% CI: 0.546–21.831), while underweight children had lower odds (OR = 0.259; 95% CI: 0.041–1.638). However, these associations were not statistically significant after adjusting for other factors (Table 2), indicating that BMI may be linked to caries risk in a nonlinear manner.

## Discussion

The combined impact of the COVID-19 pandemic and the prolonged civil war has profoundly undermined human development in Syria, leading to significant disruptions in healthcare infrastructure and a marked rise in infant mortality [33, 34]. Oral health has been particularly affected, as access to dental services and health education has become severely limited [35]. While several studies have documented the prevalence of dental caries, gingivitis, and dental trauma among Syrian children during the conflict [9, 25, 36, 37], evidence regarding the association between body mass index (BMI) and dental caries in this population remains limited.

This study is the first to explore dental caries prevalence and its association with BMI among Syrian children aged 6–9 years since the onset of the conflict in 2011, using a population-based sampling approach. These findings revealed a high prevalence of dental caries, with 85.5% of the 462 children having at least one decayed tooth, and an average DMFT+dmft score of 4.53 ± 2.79. This prevalence is lower than that reported by Alshayeb and Dashash (91%) [37] and Misrabi et al. (90%) [26], but higher than findings by Al Haffar et al. (83%) [25] and Ballouk and Dashash (79.1%) [8]. These differences may be due to variations in age groups, sample sizes, study years, and methodologies.

Evidence from comparable international studies demonstrates notable variability in the prevalence of dental caries across different populations. Al-Malik et al. reported an exceptionally high prevalence of 96% among Saudi children aged 6–7 years [38], whereas Raadal et al. documented a considerably lower rate of 67% among children aged 7–8 years [39]. Studies from Europe and Asia also reflect this diversity, with prevalence reaching 85% among Romanian children aged 6–8 years but only 61% in similarly aged children in China [40, 41]. Importantly, Yassen and Gasgoos emphasized the interplay between nutritional status and oral health, finding a significant association between excess weight and dental caries among Iraqi schoolchildren [42]. In the Syrian context, the prevalence observed in this study (85.5%) falls at the upper end of this spectrum, reflecting both regional patterns and the additional burden imposed by the ongoing conflict, which has disrupted dental services and reduced access to care [43]. These contextual factors likely contribute to the high caries rates observed among Syrian schoolchildren.

Regarding socioeconomic status, children from low-income families were 19.096 times more likely to develop dental caries than those from high-income families, a finding consistent with previous research [18, 44]. This strong association likely reflects the interplay of multiple determinants, including poor living conditions, cultural influences, psychosocial stress, and early-life adversity, all of which negatively affect health outcomes.

Limited financial resources restrict access to essential goods and services that support both oral and general health. In addition, social norms and behaviors shape health-related choices, while stress arising from adverse life events may further promote unhealthy practices. Unfavorable conditions during pregnancy and early childhood can also predispose individuals to greater disease risk later in life [45]. These mechanisms help explain the high prevalence of dental caries observed in our cohort of Syrian schoolchildren, particularly among those from low-income families, highlighting the critical influence of socioeconomic disparities on oral health in conflict-affected settings.

Unhealthy dietary habits, including skipping breakfast and consuming fewer than the recommended five daily servings of fruits and vegetables, have been consistently associated with higher dental caries prevalence [46–48]. Such behaviors may increase sugar intake frequency, reduce protective nutrients, and disrupt dietary balance, emphasizing the need to promote healthy eating habits as part of caries prevention. However, children who frequently consumed sugar-free foods had a significantly lower prevalence of caries (OR = 0.117; 95% CI: 0.046–0.297, *P* = 0.001).

Dental caries is a multifactorial disease primarily influenced by dietary sugar intake and the presence of dental biofilm. The pathogenesis involves oral pathogens within dental plaque metabolizing fermentable carbohydrates, especially sugars, into acids that demineralize tooth enamel and dentine. In addition to acid production, these pathogens release enzymes that degrade the protein matrix of the tooth, further promoting decay. Sugars are abundant in common foods such as bread, confectionery, sugary beverages, and fruits. An unhealthy diet often results in excessive sugar intake. Other dietary components beyond sugar may also contribute to caries development [49, 50]. In the context of Syrian schoolchildren, limited access to healthy foods and reliance on sugar-rich, energy-dense products during the ongoing conflict may exacerbate both excessive sugar intake and caries risk, emphasizing the need for targeted nutritional interventions as part of oral health promotion.

BMI, a key health indicator for adults and children [51], showed that 63.3% of children fell within the normal range. Among those with abnormal BMI classifications, most were classified as overweight, consistent with the findings of Zamlout et al. [13].

However, the observed increase can likely be attributed to a combination of interrelated determinants. The global obesity epidemic has been extensively documented, with contemporary studies highlighting a rapid escalation in both overweight and obesity prevalence worldwide [52]. In addition, dietary transitions toward high-carbohydrate and energy-dense foods have further exacerbated this trend [13]. Furthermore, the deterioration of socioeconomic conditions, particularly in developing countries, has been consistently associated with higher rates of overweight and obesity [53]. While these factors are not entirely discrete, their cumulative impact appears to have contributed substantially to the rising prevalence of overweight.

The relationship between BMI and dental caries has been explored in multiple studies [15, 18, 51, 54–56]. For instance, a systematic review by Paisi et al. reported a positive association between BMI and dental caries [54]. In contrast, Kotha et al. found that children with low BMI had a higher prevalence of dental caries compared to those who were overweight or obese [51]. Other studies, however, reported no significant association between BMI and dental caries [20, 57]. Overall, three systematic reviews concluded that there is no strong or consistent evidence supporting a clear relationship in this area, highlighting the need for further research [58–60].

This study identified a significant association between body mass index (BMI) and dental caries. Although multiple logistic regression did not reveal statistically significant differences across BMI categories, overweight children exhibited higher odds of developing caries (OR = 3.452), whereas underweight children showed substantially lower odds (OR = 0.259) relative to obese children. These findings indicate a complex, context-dependent relationship between nutritional status and caries risk, suggesting that both excessive and insufficient body weight may influence oral health outcomes through behavioral, dietary, and metabolic pathways.

Children today are increasingly exposed to obesogenic environments, where the accumulation of excess weight occurs with relative ease, whereas the maintenance of a healthy lifestyle requires deliberate and sustained effort. At the individual level, obesity has been strongly associated with unhealthy dietary behaviors, including frequent consumption of energy-dense and processed foods, as well as sugar-sweetened beverages, in combination with physical inactivity, prolonged screen exposure, and insufficient sleep [61]. Importantly, these dietary and behavioral patterns not only predispose children to obesity but are also closely linked to an elevated risk of developing dental caries.

The frequent intake of fermentable carbohydrates, particularly sugars, contributes to both increased caloric intake and the demineralization of tooth enamel, thereby serving as a shared risk factor for both conditions [62]. Furthermore, socioeconomic and environmental constraints often limit access to healthy food choices and promote reliance on inexpensive, high-calorie, sugar-rich products, which exacerbate the dual burden of obesity and poor oral health [63–65].

To the best of the authors’ knowledge, this is the first study examining the association between BMI and dental caries in Syrian schoolchildren, providing valuable data for monitoring obesity trends and understanding oral health risk factors in this population. The findings emphasize the importance of public health strategies targeting overweight and obesity, alongside nutritional and oral health promotion, particularly in post-conflict settings.

Although this study did not evaluate the effectiveness of specific interventions, the findings underscore the importance of developing evidence-based strategies to address shared risk factors for both dental caries and overweight. Previous reviews suggest that school-based programs may improve oral hygiene and dietary behaviors, yet their effects on reducing caries or obesity remain limited and inconsistent [66, 67]. Identifying effective and sustainable interventions remains a significant challenge in public health, particularly in resource-constrained and crisis-affected settings. Nonetheless, integrated school- and community-based approaches that combine health education, parental engagement, and improved access to nutritious foods may represent a practical framework for promoting both oral and general health among Syrian schoolchildren.

This study has several limitations. The sample was limited to 462 schoolchildren from Damascus, which may reduce generalizability. Restricting the population to schoolchildren may not represent other regions or socioeconomic groups. Caries assessment using WHO criteria at the dentin level may underestimate early lesions. Important factors such as saliva composition, detailed dietary habits, and physical activity were not assessed. Furthermore, shared risk factors for BMI and dental caries, including age, diet, oral hygiene, and socioeconomic status, were not simultaneously analyzed. Future research should address these limitations to provide a more comprehensive understanding of the determinants of both conditions.

## Conclusion

Within the limitations of this study, a high prevalence of dental caries (85.5%) was observed among Syrian schoolchildren aged 6–9 years, with no significant variation by gender or age group. In contrast, caries prevalence was significantly higher among children attending public schools and those from low socioeconomic backgrounds.

Dietary behaviors emerged as important determinants, with low consumption of fruits and vegetables markedly increasing caries risk, while higher intake of sugar was likewise associated with greater prevalence. Oral hygiene practices, particularly tooth-brushing frequency, also demonstrated a significant influence on caries occurrence.

Furthermore, Body Mass Index (BMI) exhibited a significant, nonlinear association with dental caries: overweight children experienced the highest prevalence, whereas underweight children had the lowest. These findings suggest that both undernutrition and overnutrition may contribute to caries risk through distinct pathways.

Although not evaluated in this study, multi-level interventions such as school-based initiatives, parental counseling, and community engagement may help address shared risk factors and consequently reduce the burden of dental caries. Nonetheless, evidence regarding their effectiveness remains limited, especially in crisis-affected contexts. Future longitudinal and interventional studies are warranted to better understand these associations and to develop evidence-based strategies for oral health promotion in Syrian children. However, these findings highlight important associations between studied variables and oral health, while recognizing that causality cannot be inferred due to the cross-sectional study design.

## Data Availability

The data provided for the results presented in this study is available through the corresponding author upon request.
